# What evidence exists on the drivers, ecological and socio-economic outcomes, and distribution of hunting in Peru: a systematic map protocol

**DOI:** 10.1186/s13750-026-00386-9

**Published:** 2026-04-30

**Authors:** Jennifer Jane McFarlane, Matthew Grainger, Marion Pfeifer, Louise Mair

**Affiliations:** 1 School of Natural and Environmental Science, Newcastle University, Newcastle Upon Tyne, NE1 7RU UK; 2 Instituto de Investigación en Ecología y Conservación (IIECCO), La Libertad, Trujillo, Peru; 3 Norwegian Institute for Nature Research, Torgarden, Postbox 5685, 7485 Trondheim, Norway

**Keywords:** Defaunation, Overhunting, Wildmeat, Bushmeat, Evidence synthesis, Biodiversity conservation

## Abstract

**Background:**

Across the tropics, hunting for wildmeat is essential in supporting diets, livelihoods, and food security for millions, and is also central to many cultures. Wildmeat consumption has however increased rapidly over recent decades as human population growth has increased and threatens biodiversity. Measuring hunting impacts at global scales is challenging as hunting can vary even at local scales between localities, social groups, habitat types, motivations, hunting techniques, and governance. A national scale focus would capture local literature and give context specific evidence. This evidence would inform national and subnational policies and decision-making that would be locally acceptable and sustainable. Here, we focus on hunting in Peru, where wildlife and forest research policy is motivated in developing local economies through sustainable wildlife use management and seeks scientific knowledge to meet its needs in this area. A systematic map of the literature would provide an overview of the state of knowledge on hunting and identify research gaps, which is currently lacking and would help inform policy. This protocol describes the process for conducting a systematic map to address the following question: what evidence exists on the drivers, ecological and socio-economic outcomes, and distribution of hunting in Peru?

**Methods:**

A study is included if it presents information on hunting of at least one species and a location description. Using Spanish and English languages, relevant bibliographic databases will be searched, including Peruvian-specific databases, as well as grey literature on organisational websites. Articles will be title and abstract screened, and those meeting the criteria will have meta-data extracted. Extracted data will include location and habitat, hunting details (e.g., techniques, motivations, and governance), species hunted, and reported ecological and socio-economic outcomes and how these were measured. To identify knowledge gaps, we will map the distribution of hunting studies by region, province, and habitat type. We will also describe how hunting has been studied by summarising the frequency of species hunted, hunting characteristics, and reported ecological and socio-economic outcomes, including the associated metrics and methods used.

**Supplementary Information:**

The online version contains supplementary material available at 10.1186/s13750-026-00386-9.

## Background

Tropical rainforests cover one-third of the Earth’s land surface and are of major importance to biodiversity, home to around 62% of the globe’s terrestrial vertebrates [1]. Rainforest biodiversity provides social and economic services from raw materials, timber, and non-timber forest products. Non-timber forest products include food e.g., wildmeat and fruits, fuelwood, and medicinal plants, which directly contribute to health, nutrition, income, energy, shelter, and culture for 3.5–5.8 billion people globally [2, 3]. For many people, wildmeat is their only access to protein. Therefore, it is essential for supporting diets and livelihoods for millions. Hunting is also central to culture and lived experiences. For example, it supports spiritual connections to the land and animals [4–6], traditional ecological knowledge [7], and connections to ancestral traditions, all of which allow for expression of cultural identity [8].

Consumption of wildmeat however has increased greatly over recent decades and is amongst the key threats to vertebrates globally [9]. Among the threats documented on the IUCN Red List of Threatened Species [10], hunting and trapping is considered one of the most prevalent threats to mammals and birds, potentially affecting 73% and 50% of their global ranges, respectively [11]. The drivers of this include increased human population growth, modernised hunting weapons, and greater accessibility to remote forest areas due to habitat fragmentation and increased road infrastructure [12–14]. It is estimated that 0.15 and 4.9 million tons of wildmeat are harvested annually from Neotropical and Afrotropical forests, respectively [15]. Hunting is a broad complex topic as it can occur in different settings for different purposes. These include commercial hunting for urban markets, subsistence hunting of bushmeat consumed by rural and indigenous communities, and hunting for wildlife trade products, medicine, and pets [16]. Due to high demand and rapid increase of consumption, hunting has likely become unsustainable in many areas [17, 18].

Hunting, in the context of wildmeat extraction, typically targets medium to large sized mammals, such as large herbivores, primates, and large sized birds to maximise meat returns relative to capture effort [19–21]. Under hunting pressure, these species are more vulnerable to population declines due to their life history traits. Their longer generation lengths and smaller litter or clutch sizes mean that populations take longer to recover following harvesting [21, 22]. Overhunting can lead to loss of species and subsequently ecosystem functional roles, including predation, browsing, seed depredation, and seed dispersal. This can cause cascading effects on species community structures e.g., increased rodent and mesopredator numbers [23, 24], as well as forest structure, including increased plant density and decreased plant diversity [25, 26], and altered seed dispersal and tree growth dynamics [26, 27]. This can lead to the “empty forest syndrome” where forests are structurally intact but functionally impoverished [28–30].

Hunting drivers vary across continents due to differing hunting contexts. In Asian forests, human population density is very high and high forest degradation and fragmentation combine with strong international wildlife trade [31–34]. Adoption of modern, highly efficient guns and snares has increased harvest efficiency and reduced skill required, leading to substantial population declines in the last three decades [12]. In Africa, human population density and forest degradation are lower than Asia, but the number of species involved in wildlife trade is similarly high [31–34]. In Africa, hunting is more affordable than livestock grazing, and the use of snares is widespread. Snares are simple, durable, and cheap, which has allowed hunting to increase rapidly as many can be deployed over a greater area [13]. In South America, human population density is much lower [34], the Amazon forest is relatively intact although the Atlantic forest is heavily fragmented [31, 32]. Wildlife trade involves fewer species, but the pet trade for amphibians remains high [33]. Hunting technology includes shotguns and bow and arrows, however, in some areas hunting is believed to have reached unsustainable levels [35, 36].

Hunting also varies across social groups who differ in social and economic backgrounds, which may influence hunting motivations and the species targeted. Differences in hunting practices between indigenous and non-indigenous groups are reported in the literature. For example, in Afrotropical forests, non-indigenous groups using guns and snares for commercial hunting have been reported to extract 27 times more bushmeat than the Baka/Aka/Ef indigenous groups, who use bows and arrows for subsistence hunting and target a narrower range of species [37]. Asian forests, home to the largest number of indigenous peoples in the world, are characterised by a variety of hunting technologies used by these groups such as spears, blowpipes, traps, and bows and arrows, each suited to capturing different species [38]. However, urban wealth is a major hunting driver due to the luxury wildlife trade market, traditional Chinese medicine, and the live pet trade [39, 40]. In the Neotropics, hunter profiles differ amongst communities engaged in subsistence hunting, with cultural differences between ethnolinguistic groups [8]. In addition, traditional hunting technology may be more sustainable due to its lower efficiency compared to shotguns [41, 42].

Sociocultural aspects are also key to hunting purposes. Hunting activity promotes learning social norms, acquiring knowledge of the forest and skills, and forming bonds with fathers e.g., among the BaYaka indigenous group in Africa [43]. Hunting can also function as rituals and a way to show bravery and provide trophies which symbolise power. For example, the BaTonga of the Congo Basin carry out “royal hunts” and make food offerings in ancient ceremonies [44]. Hunting is also embedded in cosmology and worldviews. For example, in Matsigenka mythology in Peru, the Harpy eagle spirit, once believed to have walked the earth in human form, taught shamans the knowledge and skills of hunting [6]. Social norms may also define the choice of hunting tools. For example, the Sharanahua in Peru use bows and arrows for animals considered appropriate for consumption, whereas spears are used for animals associated with danger and confrontation, such as jaguars [45]. Similarly, the Huaorani in Ecuador use blowguns for arboreal prey but spears for larger terrestrial animals, reflecting differences in hunting context and the social significance of technological choices [46]. In addition, sharing wildmeat functions as strengthening social bonds as well as maintaining food security among the community [47]. In Amazonian societies, food taboos and hunting rules form structures in resource use and are based on social and spiritual relations between human beings and animals [48].

Sociocultural factors also interact with ecological dimensions of hunting. A social group may have hunting taboos and beliefs which will drive hunter species preferences and motivations [49]. For example, in Asia, Muslim communities will not consume wild pigs, reducing hunting motivations for the species in those areas [12]. Moreover, across the tropics, animistic beliefs, the belief that an animal, plant or object has a soul and may be benevolent or malevolent, among some cultures leads to avoidance in hunting some species either entirely or at certain times in one’s life [50–52]. These beliefs in turn will drive differences in species harvest rates which may affect species community composition and could ultimately affect ecosystem function and forest structure. Another example is the Maijuna community in Peru, who hold traditional beliefs about why animals regularly visit mineral licks (a primary hunting location) which are linked to the Maijuna story of the creation of the first tapir [53]. The distribution and attractiveness of these salt licks to certain species, particularly large mammals, may influence the species hunted and harvest rates, and in turn shape the spatial distribution and composition of species. In addition, different hunting technology choices result in differing biomass harvests, e.g., the use of dogs [54], stalking vs. opportunistic [55], and shotguns vs. bow and arrows [56].

Hunting pressure impacts have been assessed using a wide variety of indicators. These include changes in abundance [57], occupancy [58, 59], density [42], functional composition [36, 60], and biomass [61]. Data collection for these indicators varies and are associated with different levels of uncertainties. For example, camera traps excel in capturing cryptic and elusive species but rely on unbiased placement [62]. Line transects can under detect species [63], and interviews rely on participants recalling information over a long time period [64] which could lead to under or overestimates of hunting pressure impact. Furthermore, sampling designs can be confounded if indicators are measured without accounting for confounding variables such as habitat loss, fragmentation, and deforestation, which are known to synergistically interact with hunting [65]. Finally, sustainability indices do not account for spatial heterogeneity of species and there are uncertainties in parameter estimates such as rate of population increase [66, 67].

Defaunation indices have been used at global and continental scales to map hunting pressure impact. These estimate species abundance in pairwise studies of hunted and non-hunted sites [28] or compare observed (contemporary) and expected (historical) species richness [68]. These indices are then used to project over a map an extrapolation or interpolation of changes in species assemblages attributed to hunting variables. These variables can include distance to roads (hunter access), human population density, protected area status (law enforcement), and species identity or traits. However, data feeding into these indices have not been collected systematically, introducing spatial bias in models. The limited breadth of variables used in the modelling restricts predictive capability across large spatial scales, as it does not account for socio-economic factors that shape hunting motivations and techniques, or their interaction with local patterns of biodiversity and habitat [69, 70]. Resulting maps are thus likely unreliable at local and landscape scales, as they need to be based on robust evidence [69].

Accounting for local context when estimating and mapping hunting activity and designing suitable interventions is a complex challenge. For example, at national and subnational scales, governance, hunting motivations, and techniques interact to shape hunting patterns and associated ecological and socio-economic outcomes. Such complexities are difficult to incorporate in meta-analyses [71]. Addressing this complexity requires a systematic assessment of the available evidence on the interactions between hunting, biodiversity, and socio-economic variables, accounting for local context. Systematic mapping provides a structured approach to collate and describe this evidence base, helping to identify knowledge gaps and inform context-specific decision-making. This is more challenging in countries where hunting is prevalent and interacts with a large diversity of species [8, 72]. Such countries are typically tropical, biodiversity rich nations with deep-rooted hunting and wildmeat consumption traditions and cultures, which differ in space [44, 73, 74] and locality [69]. These local studies are often published in the country’s official language(s) and country specific databases. For example, non-English studies typically provide local information from field practitioners, but language barriers may prevent publication to international journals, and so they may be published in a country-specific database [75]. Moreover, non-English studies can provide information in areas where there is no English language evidence [76]. A national-scale focus would therefore enable the inclusion of local literature and searches in the country’s official language(s), providing more context-specific evidence on variation in hunting activity, associated motivations and techniques, and reported ecological and socio-economic outcomes.

## The Peruvian hunting landscape

In Peruvian forests, hunting is a widespread legal activity and is increasingly being monitored locally in participatory programs and community initiatives in many areas [41, 77, 78]. Peruvian forests are still largely intact compared to tropical regions elsewhere [79], reducing confounding effects of forest fragmentation that can mask patterns in the evidence base [62]. Peru retains one of the most faunally intact biomes, providing an opportunity to describe hunting across a wide range of vertebrate functional groups. As in other pantropical landscapes, Peru has many social groups with potentially different hunting drivers, and hunting varies hugely with habitat type, hunting techniques used, and socio-economic contexts [80].

A systematic map at Peruvian national scale would be directly relevant to the government informing actions on locally needed conservation or research that would close existing knowledge gaps and describe species hunted, the variation of legal and illegal hunting, the local socio-economic context, and associated hunting motivations and techniques. This is critical data for The Peruvian National Forestry and Wildlife Service’s (SERFOR) National Plan of forest and wildlife research strategy (2020–2030). It aims to promote the conservation and sustainable use of the forest and wildlife [81]. To do this, it has regulatory frameworks and national forest and wildlife policies for reconciling conservation with socio-economic needs. Within the strategy, key research priorities include the identification and management of wildlife species (and species with economic potential), illegal hunting for consumption, anthropogenic activities affecting wildlife in critical habitats, and the socioeconomic interactions of wildlife on communities [81]. A key motivation underpinning these priorities is to enable the development of local economies via sustainable wildlife use management. However, one of the main problems identified by stakeholders (public institutions, experts, industrial entrepreneurs, academia, research institutions, international cooperations from nine Peruvian regions) is that there is scarce scientific knowledge and technologies that can meet the needs of the forestry and wildlife sector. The strategy therefore aims to support the adoption of new processes and technologies to improve productivity and competitiveness [81].

## Stakeholder engagement

We disseminated a summary of this systematic map containing the abstract, research questions, PECO structure, database sources, and meta-data codes from the literature to experts via email, remote, and in-person discussions. We received six responses ranging in diverse perspectives from global (IUCN), governmental (Ministry of Environment), national and regional organizations, universities, and independent experts in environmental conservation (San Diego Zoo Wildlife Alliance Peru, Conservación Amazónica). They found the research question to be relevant and useful for management, policy, and authorities and suggested grey literature sources.

### Objective of the review

Using Peru as a case study this systematic map aims to assess the extent of knowledge on the drivers, scales, and characteristics of hunting at Peruvian national scale and to identify interactions between them that shape ecological and socio-economic outcomes. The focus is on characterising hunting e.g., hunting motivations, techniques, hunter species preferences etc., and to describe existing evidence on ecological and socio-economic outcomes of hunting.

### Primary question

What evidence exists on the drivers, ecological and socio-economic outcomes, and distribution of hunting in Peru?

### Primary question components

The primary question components were developed using an adapted PECO structure [82]. We used Population (P), Exposure (E), and Outcome (O) components. Comparator (C) is not applicable because it is used for meta-analysis reviews aiming to study exposure effect sizes. If an article presents ecological and/or socioeconomic outcomes of hunting these will also be captured. Many papers do not present hunting outcomes but do present information on hunting activities, species hunted, and community background descriptions which is valuable information for the review and will still be included if they report the minimum criteria. The minimum criteria are at least one hunted species and a location description. The question components are therefore:

- Population: Terrestrial mammals, birds, reptiles, and amphibian species in Peru.
- Exposure: Hunting in Peru.
- Outcome: any ecological and/or socioeconomic aspect of hunting (if reported).

### Secondary questions

To characterise hunting activity and assess the outcomes and the distribution of hunting in Peru, we will extract meta-data from the literature that is guided by the following secondary questions:

- What is the distribution of the hunting literature in Peru, and does it differ across regions, provinces, and habitat type?
- Which species are hunted, and which are hunters’ preferred species?
- What types of ecological outcomes related to hunted species have been reported in the literature and how have these outcomes been measured?
- What types of socio-economic outcomes related to hunting have been reported in the literature and how have these outcomes been measured?

#### Methods

This protocol complies with the RepOrting standards for Systematic Review Syntheses (ROSES) for systematic map protocols (Additional file 1) and follows the Collaboration for Environment Evidence (CEE) Guidelines and Standards for Evidence Synthesis in Environmental Management [83].

#### Searching for articles

##### Search string

Based on the Population (P) and Exposure (E) components and 13 benchmark papers identified through scoping searches and prior knowledge, key search terms were extracted from titles and abstracts (Additional file 2). Outcome terms were not included in the search strategy to ensure broad capture of relevant studies but will be applied during data extraction. We then identified search term synonyms using a thesaurus. We included country and region names in the search terms to restrict studies to Peru. We then added the Boolean operator “OR” to combine the search terms and synonyms. Each question component search string was then combined with the Boolean operator “AND” (Table 1). Both English and Spanish language combinations of different synonyms were tested in SCOPUS for optimal sensitivity and specificity against the benchmark papers (Additional file 3). An asterisk (*) is used as a wildcard for some words (i.e., it is used to represent any number of characters at the end of the word), for example hunt* will also include hunting, hunted, and hunter. The final search string was then adjusted according to the search capabilities and syntax of other databases (Additional file 3).

##### Estimating the comprehensiveness of the search

The benchmark articles described above were used to test search sensitivity in SCOPUS. Search strings were iteratively refined to ensure retrieval of all benchmark studies (Additional file 3).

##### Database searches

The following databases will be searched using the search string described above:

1. SCOPUS.
2. Web of Science Core Collection - Indexes: SCI-EXPANDED, SSCI, AHCI, CPCI-S, CPCI-SSH, ESCI.
3. SciELO Citation Index.
4. CAB Abstracts.
5. ProQuest Natural Science Collection.
6. LA Referencia - The Latin American Network for Open Science. An open access aggregated repository of Latin American and Spanish theses (undergraduate, masters, and PhD).

Subscription databases will be accessed via Newcastle University.

Additional Peruvian-specific databases will also be searched:

1. Renati digital database (SENEDU) - A collection of Institutional repositories containing works from Peruvian universities and theses obtained abroad.
2. ALICIA (CONCYTEC) - The National Digital Repository of Science, Technology and Innovation with open access to the national scientific literature.

The search results from these databases, the following grey literature, and web-based targeted search will be updated to include the year 2025.

##### Specialist search for grey literature

In addition to thesis works from LA Referencia and Renati, we will search organisations, NGOs, and Peruvian governmental departments for relevant reports (Table 2). Simplified strings, or a keyword, will be used and changed iteratively depending on result relevance and website search capabilities. Titles and summary descriptions will be pre-screened within the website. Relevant articles will then be full text screened in Rayyan software. Final search words will be recorded with website links and the number of relevant results retrieved in the final systematic map.

##### Targeted searches

We will also conduct a targeted search in Google Scholar search engine using a simplified search string. We will screen the first 200 results, organised by relevance. For efficiency, only titles within Google Scholar will be screened. We will also engage with stakeholders from relevant government departments and NGOs to request any relevant literature that they may have.

##### Assembling and managing search results

Search results will be exported to Endnote and duplicates will be removed using combinations of duplicate removal preferences (Additional file 4). This was tested using a subset of search results from SCOPUS and Web of Science core collection and checked using Rayyan’s duplicate similarity tool. This allowed for additional combinations of Endnote duplicate removal preferences to be made until all similarity scores were below 95%. Some duplicates appear in Spanish and English; therefore, a comparison of all studies with the same author names will also be necessary in Endnote.

#### Article screening and study eligibility criteria

##### Screening process

We will conduct a two-stage screening process, (1) title and abstract, and (2) full-text using Rayyan software. If the reviewer is unsure of study eligibility or there is not enough information to determine inclusion, the study will pass to the full-text stage. The primary reviewer will screen 100% of the results, and for consistency checking an additional two reviewers will each screen 50% of results in both two-stage processes. A full archive of excluded studies with reasons will be published as an additional file alongside the review in accordance with ROSES standards. For additional consistency checks, at each stage a Cohen’s Kappa coefficient will be calculated from a random subset of results to test agreement between reviewers. A threshold of K ≥ 0.6 will be considered acceptable. Where agreement falls below this level, reviewers will discuss discrepancies to reach consensus and clarify application of the criteria. If consensus is not achieved, a third reviewer (project supervisor) will be consulted.

The primary author declares that one of their previous publications is expected to be considered in the review. To minimise potential bias, reviewers will not be involved in screening decisions or data coding for studies they have authored. These decisions will be conducted by secondary reviewers.

##### Eligibility criteria

Literature will be screened using inclusion and exclusion criteria (Table 3). These criteria were developed through pilot testing on a subset of search results from SCOPUS and Web of Science core collection in Rayyan. Criteria were adjusted when there were doubts or gaps found. The scope of this study is terrestrial hunting; therefore, species associated with fishing activities are excluded. Thus, all marine vertebrates and freshwater vertebrates that only live in water (e.g., river dolphins and manatees) are excluded. Amphibians and freshwater reptiles that use the terrestrial environment are included as these are a key protein source for some forest communities. Invertebrates are excluded as they are associated with collection and gathering activities instead of hunting. Studies will not be restricted by publication year, but studies on hunting activity before the Spanish conquest (1532) will be excluded as this is before many communities turned to sedentism under Spanish rule and missionaries (i.e., when hunting around a settlement became widespread and continues today, with the exception of indigenous groups in isolation). Changes from nomadism to sedentism meant that resource use became concentrated around settlements locally over long-time scales, as is current resource use today. Primary literature in the format of scientific publications, student theses (BSc, MSc, PhD), book chapters, reports, and grey literature publications will be included. Reviews will be excluded.

#### Data coding strategy

At full text screening, all literature will be read by the primary reviewer. Two secondary reviewers will read 50% each. Data from text, figures, and tables will be entered using codes in an Excel coding sheet with definitions provided (Additional file 5). This coding sheet was developed using a subset of studies identified as eligible during pilot testing. The complete data extraction code sheet will be published alongside the review as an additional file. Cohen’s Kappa coefficient will be calculated on a subset of studies to assess agreement between reviewers on the categorical meta-data to be extracted (K ≥ 0.6). Where agreement is < 0.6, discrepancies will be resolved following the same procedure as in screening, with clarification of code definitions where necessary. Meta-data extraction will encompass the following:

1. Study characteristics
  Publication details.
  Study location and habitat type (location name, coordinates, habitat description).
  Study purpose, sample site and participant number.
2. Hunting details
  Hunting technique (shotgun, bow and arrow, trap, net, spear, snare, dog).
  Hunting motivations (subsistence, commercial, cultural, ornament, pet trade, sport & recreation, medicinal, human-wildlife conflict, opportunistic).
  Hunting frequency (occasionally, weekly, monthly seasonally).
  Hunting group size (solo, pair, group of 3 or more).
  Community type (indigenous, non-indigenous).
  Community comments (description, e.g. ethnic group name).
  Settlement type (rural, urban).
  Governance (legal, illegal).
  Management type (community management, government management, private management).
3. Species and biodiversity
  Hunted species.
  Hunter species preferences.
4. Outcomes
  Outcome type (ecological, socio-economic).
  Outcome category.
  Outcome subcategory.
  Metric.
  Method.

The study purpose will be extracted along with region and province. The reported coordinate system, coordinates, and elevation will be extracted, and the geolocation accuracy scored on a scale of 1 to 5 (see Additional file 5 sheet 2 for definitions). Coordinates will be converted into UTM to extract province name if not reported. A preliminary screening indicates that few studies record the GPS locations of where species have been caught, making it difficult to assign hunted wildlife to habitat type. Therefore, for each hunted species, we will extract the habitat type as reported by authors and species habitat preferences from the IUCN Red List of Threatened Species database using their habitat classification scheme [84]. Descriptions of study characteristics will include the number of sample sites and/or the number of participants. We will extract community type as either indigenous or non-indigenous. These groups can be separated into subcategories such as campesinos, colonists, criollos, mestizos, and ribereños for non-indigenous groups [85], and indigenous groups can be separated by ethno-linguistic groups such as Pano and Arawak [8]. Within these groups there are many overlapping social and economic factors therefore to capture nuances in describing communities we have two additional data codes: community type comment, which will capture the ethnic group and/or language described; and settlement type, which will capture if the community are in a rural or urban setting. Governance (legal or illegal hunting according to Peruvian state law), and management type will also be extracted.

We will extract hunting motivation, defined as the primary motivation(s) for hunting. For those studies related to the wildlife trade (e.g. studies recording harvests and species hunted from markets), hunting motivations will be classed as commercial hunting. The selling motivation (i.e. sold for food, medicine, decoration, pet etc.) will not be considered as this is a secondary purpose to do with trade and not hunting. For ecological and socio-economic outcomes, we will extract the following meta-data: outcome type (ecological or socio-economic), outcome category and subcategory (as defined in Additional file 5), the metric used to quantify the outcome, and the method used to measure or assess the outcome. For example, an ecological outcome may be classified as category: population status (subcategory: abundance), with a metric of number of individuals per km² and measured using transect surveys. A socio-economic outcome may be classified as category: income and livelihoods (subcategory: cash income), with a metric of monthly income (USD) and measured using household surveys.

**Table 1:** Search terms corresponding to PECO components and final search string

PECO structure components	Search terms
Population	English: amphibian* OR reptile* OR chelonia* OR bird* OR cracid* OR mammal* OR “large vertebrate*” OR wildlife OR “wild life” OR species Spanish: anfibio* OR reptil* OR quelonio* OR ave* OR crácido* OR mamífero* OR “grandes vertebrados” OR “vida silvestre” OR “vida salvaje” OR especie*
Exposure	English: hunt* OR overhunt* OR trap* OR bushmeat OR “bush meat” OR poach* OR game OR wildmeat OR “wild meat” Spanish: caza* OR cacería OR “cazar furtivamente” OR trampa* OR atrapan OR “carne salvaje” OR “carne de animales silvestres”
Locator	Peru* OR “Madre de Dios” OR Amazonas OR Ucayali OR Loreto OR “San Martín” OR Huánuco OR Pasco OR Junín OR Huancavelica OR Ayacucho OR Apurímac OR Cuzco OR Puno OR Tacna OR Moquegua OR Arequipa OR Ica OR Lima OR Ancash OR “La Libertad” OR Cajamarca OR Lambayeque OR Piura OR Tumbes
Search string developed in SCOPUS	TITLE-ABS-KEY (hunt* OR overhunt* OR trap* OR bushmeat OR “bush meat” OR poach* OR game OR wildmeat OR “wild meat” OR caza* OR cacería OR “cazar furtivamente” OR trampa* OR atrapan OR “carne salvaje” OR “carne de animales silvestres”) AND (amphibian* OR reptile* OR chelonia* OR bird* OR cracid* OR mammal* OR “large vertebrate*” OR wildlife OR “wild life” OR species OR anfibio* OR reptil* OR quelonio* OR ave* OR crácido* OR mamífero* OR “grandes vertebrados” OR “vida silvestre” OR “vida salvaje” OR especie*) AND (Peru* OR “Madre de Dios” OR Amazonas OR Ucayali OR Loreto OR “San Martín” OR Huánuco OR Pasco OR Junín OR Huancavelica OR Ayacucho OR Apurímac OR Cuzco OR Puno OR Tacna OR Moquegua OR Arequipa OR Ica OR Lima OR Ancash OR “La Libertad” OR Cajamarca OR Lambayeque OR Piura OR Tumbes)

**Table 2:** List of organisations, government websites, and NGOs to search for additional grey literature

Organisation	Links (accessed 17/07/2025)
IUCN	https://iucn.org/search-website
WWF	https://www.worldwildlife.org/search; https://www.worldwildlife.org/peer-reviewed-publications
San Diego Zoo Wildlife Alliance (SDZWA)	https://science.sandiegozoo.org/publications; https://science.sandiegozoo.org/resources/saving-species; https://science.sandiegozoo.org/search/node/hunting%20AND%20Peru
Frankfurt Zoological Society Peru (FZS)	https://peru.fzs.org/publicaciones/publicaciones-cientificas/
Wildlife Conservation Society Peru (WCS)	https://peru.wcs.org/es-es/WCS-Peru/Publicaciones.aspx
Peruvian Amazon Research Institute (IIAP)	https://repositorio.iiap.gob.pe/simple-search?filterquery=true&filtername=has_content_in_original_bundle&filtertype=equals
Peruvian National Parks Service (SERNANP)	https://sis.sernanp.gob.pe/biblioteca/
National Forest and Wildlife Service (SERFOR)	https://repositorio.serfor.gob.pe/simple-search?location=SERFOR%2F215&query=carne+silvestre&rpp=10&sort_by=score&order=desc
National Environmental Information System (SINIA)	https://sinia.minam.gob.pe/search?content_type=documentos
Ministry of the Environment (MINAM)	https://www.gob.pe/institucion/minam/informes-publicaciones
The Nature Conservancy	https://www.nature.org/en-us/what-we-do/our-insights/reports/
Alliance for a Sustainable Amazon	https://www.sustainableamazon.org/publications
Amazon Conservation Association	https://acca.org.pe/publicaciones/documentos-tecnicos/
Fauna Forever	https://www.faunaforever.org/publications

**Table 3:** PECO framework question elements and eligibility criteria to be used for literature screening

PECO structure components	Question element	Criteria
Population	Terrestrial mammals, birds, and freshwater reptiles and amphibian species in Peru	Inclusion: terrestrial vertebrate species, and freshwater reptiles and amphibians that use the terrestrial environment in Peru Exclusion: all marine vertebrates, domesticated vertebrates, all freshwater fish, river dolphins and manatees, completely aquatic species, invertebrates
Exposure	Hunting	Inclusion: primary literature that include information on hunting. The minimum information required is a location description and at least one hunted species (see additional criteria below for formats accepted) Exclusion: zooarchaeological and palaeontology studies of hunting in the past (pre-Spanish conquest before 1532), studies solely on species distribution/inventory/other metrics without a hunting description, and studies on predation i.e., animals hunting other animals/species hunting behaviour, and fishery studies
Outcome	Ecological and/or socio-economic outcomes of hunting	No eligibility criteria applied: If reported, any ecological and/or socio-economic outcomes related to hunting will be recorded using defined categories and subcategories (Additional file 5)
Additional criteria	Format of the result	Inclusion: scientific publications, student theses (BSc, MSc, PhD), book chapters, reports, grey literature publications Exclusion: reviews, journalism e.g. news and magazine articles, manuals, grant proposals, opinion pieces, presentations, conference proceedings, formats with insufficient information e.g., faulty links, inaccessible works, or there is no publication associated with the result

#### Study mapping and presentation

We will present a narrative synthesis to describe the distribution of evidence across hunting studies in Peru, including species and their habitat types, hunting motivations and techniques, community characteristics, and reported ecological and socio-economic outcomes. For visualisation of the distribution of the literature, we will present a geographic map showing the number of studies per region and province, and a bar chart of the number of studies per habitat type. Ecological and socio-economic outcomes will be summarised using bar charts to describe the distribution of outcome categories and subcategories, and the associated metrics and methods used to measure them. Hunting community type, motivations, and techniques will be summarised using bar charts. The species hunted and reported species preferences will be presented using a treemap diagram. The workflow of the review will be presented in a ROSES flow diagram, and the results will be published.

## Supplementary Information

Additional file 1. ROSES for Systematic Map Protocols. The ROSES checklist.

Additional file 2. Benchmark papers and keywords. The list of benchmark papers, and keyword and synonym identification.

Additional file 3. Search string test in SCOPUS. Search string test in SCOPUS and the final search string to be used in each database.

Additional file 4. Endnote deduplication steps. The duplication preference adjustment settings used in Endnote to remove duplicates.

Additional file 5. Codes and definitions. The data extraction codebook.

## Data Availability

All data generated or analysed during this study are included in this published article and its supplementary information files.
