# Supplementary material for: What evidence exists on the drivers, ecological and socio-economic outcomes, and distribution of hunting in Peru: a systematic map protocol
Source: Environ Evid. 2026 Apr 30;15:7. doi: 10.1186/s13750-026-00386-9 (PMC13285517; doi:10.1186/s13750-026-00386-9)
Supplement: Supplementary file 2 — Additional file 2. Benchmark papers and keywords. The list of benchmark papers, and keyword and synonym identification. [file 13750_2026_386_MOESM2_ESM.docx]

1. Benchmark articles used to identify key search terms
2. Bodmer, R. E., Eisenberg, J. F., & Redford, K. H. (1997). Hunting and the Likelihood of Extinction of Amazonian Mammals. *Conservation biology*, *11*(2), 460-466. https://doi.org/10.1046/j.1523-1739.1997.96022.x
3. Costa, J. F., Ríos-Choronto, R., Peña-Candia, L. Z., & Simões, E. (2018). Aves y mamíferos silvestres usados por pobladores del Bajo Urubamba, Cusco, Perú. *Revista Peruana de Biología*, *25*(4), 451-458. https://doi.org/10.15381/rpb.v25i4.15534
4. D’Cruze, N., Elwin, A., Perez-Peña, P. E., Vieto, R., Asfaw, A. E., & Harrington, L. A. (2024). Wildlife trade at Belén and Modelo market, Peru: defining a baseline for conservation monitoring. *Frontiers in Conservation Science*, *5*, 1464332.

https://doi.org/10.3389/fcosc.2024.1464332

1. Francesconi, W., Bax, V., Blundo-Canto, G., Willcock, S., Cuadros, S., Vanegas, M., Quintero, M., & Torres-Vitolas, C. A. (2018). Hunters and hunting across indigenous and colonist communities at the forest-agriculture interface: an ethnozoological study from the Peruvian Amazon. *Journal of ethnobiology and ethnomedicine*, *14*(54), 1-11. https://doi.org/https://doi.org/10.1186/s13002-018-0247-2
2. Gavin, M. C. (2007). Foraging in the fallows: Hunting patterns across a successional continuum in the Peruvian Amazon. *Biological Conservation*, *134*(1), 64-72. https://doi.org/doi:10.1016/j.biocon.2006.07.011
3. Gilmore, M. P., Griffiths, B. M., & Bowler, M. (2020). The socio-cultural significance of mineral licks to the Maijuna of the Peruvian Amazon: implications for the sustainable management of hunting. *Journal of ethnobiology and ethnomedicine*, *16*(1). https://doi.org/10.1186/s13002-020-00412-1
4. Gonzales Guillén, F. N., & Llerena Reátegui, G. (2014). Cacería de mamíferos en la Zona de Uso Especial y de Amortiguamiento del Parque Nacional Tingo María, Huánuco, Perú. *Revista Peruana de Biología*, *21*(3), 283-286. https://doi.org/10.15381/rpb.v21i3.10904
5. Griffiths, B. M., Bowler, M., Kolowski, J., Stabach, J., Benson, E. L., & Gilmore, M. P. (2022). Revisiting Optimal Foraging Theory (OFT) in a Changing Amazon: Implications for Conservation and Management. *Human Ecology*, *50*(3), 545-558. https://doi.org/10.1007/s10745-022-00320-w
6. Mayor, P., El Bizri, H. R., Morcatty, T. Q., Moya, K., Bendayán, N., Solis, S., Vasconcelos Neto, C. F. A., Kirkland, M., Arevalo, O., Fang, T. G., Pérez‐Peña, P. E., & Bodmer, R. E. (2022). Wild meat trade over the last 45 years in the Peruvian Amazon. *Conservation biology*, *36*(2). https://doi.org/10.1111/cobi.13801
7. Mayor, P., Pérez-Peña, P., Bowler, M., Puertas, P. E., Kirkland, M., & Bodmer, R. (2015). Effects of selective logging on large mammal populations in a remote indigenous territory in the northern Peruvian Amazon. *Ecology and Society*, *20*(4). https://doi.org/10.5751/es-08023-200436
8. McFarlane, J. J., Chacón, O. M., Arauco‐Aliaga, R. P., Braunholtz, L., Sanderson, R., & Pfeifer, M. (2024). Selective impacts of subsistence hunting on mammal communities in Manu National Park, Peru. *Biotropica*, *56*(5). https://doi.org/10.1111/btp.13367
9. Menajovsky, M. F., Espunyes, J., Ulloa, G., Montero, S., Lescano, A. G., Santolalla, M. L., Cabezón, O., & Mayor, P. (2024). A Survey of Hepatitis B Virus and Hepatitis E Virus at the Human–Wildlife Interface in the Peruvian Amazon. *Microorganisms*, *12*(9), 1868. https://doi.org/10.3390/microorganisms12091868
10. Ohl‐Schacherer, J., Shepard, G. H., Kaplan, H., Peres, C. A., Levi, T., & Yu, D. W. (2007). The Sustainability of Subsistence Hunting by Matsigenka Native Communities in Manu National Park, Peru. *Conservation biology*, *21*(5), 1174-1185. https://doi.org/10.1111/j.1523-1739.2007.00759.x
11. Keyword identification

Using benchmark papers identified through preliminary scoping searches and prior knowledge of the literature, a keywords list was generated from their titles and abstracts. Selected keywords for synonym selection (see section 3) are shown in bold.

| **Article number** | **Population** | **Exposure** | **Location** |
| --- | --- | --- | --- |
| 1 | **Mammals**  **Species**  large-bodied **mammals**  Amazonian **mammals**  **Mamiferos** Amazónicos  **Especies** | **Overhunt**ing  **Hunting**  **hunt**ing pressure  **Caza**  Presión por **caza** | **Peru**vian  Amazonian |
| 2 | **Aves**  **Mamíferos** silvestres  **Mamíferos** aprovechadas  **Mamíferos** grandes  **Especies** | **Caza**  Carne  Alimentación  Partes del cuerpo  Proteína animal  Aprovechamiento de **Especies** | **Cusco**  **Perú**  Selva amazoníca |
| 3 | **Wildlife**  **Species**  **Mammals**  **Reptiles**  **Birds**  Lowland paca  Peccaries  Caiman  River turtles  Boas  Yellow footed tortoise  Parrots  Brocket deer | **Wild meat**  Illegal wildlife trade  Live pets  Traditional medicine  Belief-based use | **Peru**  Iquitos |
| 4 | **Wildlife**  Lowland pacas  Red brocket deer  Prey | **Hunt**ers  **Hunt**ing  Livelihood strategies  **Bush meat**  Food security | **Peru**vian Amazon  **Ucayali**  Pucallapa |
| 5 | **Species**  **Vertebrate**  **Mammals**  **Wildlife** | **Hunti**ng  Sustainable harvest  Extraction events  Meat  Harvest rates  Overharvest | **Peru**vian Amazon |
| 6 | **Wildlife** populations  **Mammals**  Biodiversity | **Hunt**ing  **Hunt**ers  Traditional ecological knowledge  Mineral licks  traditional sustainable **hunt**ing | **Peru**vian Amazon |
| 7 | **Mamíferos**  **Especies** vulnerables  **Especies** **caza**das  **Mammal**  Hunted **species**  Vulnerable **species**  Dasyprocta sp.  Cuniculus paca  Pecari tajacu | **Cacería**  Métodos de **cacería**  **Hunt**ing | **Huánuco**  **Perú** |
| 8 | **Mammals**  Paca  Collared peccary  Brocket deer  Tapir  large-bodied primates | **Hunt**er  Subsistence **hunt**ing  Decision-making  Wild **game**  **Game** meat  Prey selection  Optimal foraging theory | Amazon  Northeastern Amazon  **Peru** |
| 9 | **Wildlife**  Ungulates  Rodents  Endangered **species**  Primates | **Wild meat** trade  **Bushmeat**  **Wild meat**  Wild-meat markets  Public health  Sustainability  Urban markets  Wildlife trade | Amazonia  **Peru**vian Amazon |
| 10 | **Mammal**  Large **mammal** populations  Rodents  Primates  Collared peccaries  Tapirs  White-lipped peccaries  **Wildlife** | **Hunt**ing  Timber logging  Catch per unit effort  **Hunt**ing registers  Subsistence **hunt**ing | **Peru**vian Amazon  Amazon |
| 11 | **Mammal** communities  **Species** communities  Forest **wildlife**  Carnivores  Rodents  Large herbivores  Sensitive **species** | Sustainable **hunt**ing  **Wild meat**  **Hunt**ing pressure  Local depletion | **Peru** |
| 12 | **Wildlife species**  Cuniculus paca  Tayassu pecari  Mazama americana  Wild animals | **Hunting**  Consumption of wild animals  Hepatitis B virus  Hepatitis E virus | **Peru**vian Amazon |
| 13 | **Large vertebrates**  Woolly monkey  Spider monkey  white-lipped peccary  Razor-billed currasow  Spix’s Guan  Hunted **species**  **Vida silvestre** | **Bushmeat**  Subsistence **hunt**ing  **Wild meat**  **Game**  **Cacería** de subsistencia  Explotación de carne | **Peru**  **Peru**vian Amazonia  Amazonía peruana |

1. Selected Keywords and their Synonyms

Selected keywords from the keyword identification step are shown in bold with their synonyms shown in plain text. The full list was then tested in SCOPUS for sensitivity and specificity (Additional file 3).

| **Population** | **Exposure** | **Location** |
| --- | --- | --- |
| **Mammals**  **Species**  **Wildlife**  **Reptile***  **Bird***  **Large vertebrate***  **Mamifero***  **Especie***  **Ave***  **Vida silvestre**  Amphibian  Chelonia*  Cracid*  Wild life  Anfibio*  Reptil*  Quelonio*  Crácido*  Vida salvaje | **Overhunt***  **Hunt***  **Wildmeat**  **Bushmeat**  **Game**  **Caza***  **Cacería**  Trap  Bush meat  Wild meat  Poach  Cazar furtivamente  Trampa  Atrapan  Carne salvaje  Carne de animales silvestres  Defaunation | **Peru***  **Cusco**  **Ucayali**  **Huánuco**  Madre de Dios  Amazonas  Loreto  San Martin  Pasco  Junín  Huancavelica  Ayacucho  Aprímac  Puno  Tacna  Moquegua  Arequipa  Ica  Lima  Ancash  La Libertad  Cajamarca  Lambayeque  Piura  Tumbes |
