# Supplementary material for: What evidence exists on the drivers, ecological and socio-economic outcomes, and distribution of hunting in Peru: a systematic map protocol
Source: Environ Evid. 2026 Apr 30;15:7. doi: 10.1186/s13750-026-00386-9 (PMC13285517; doi:10.1186/s13750-026-00386-9)
Supplement: Supplementary file 3 — Additional file 3. Search string test in SCOPUS. Search string test in SCOPUS and the final search string to be used in each database. [file 13750_2026_386_MOESM3_ESM.docx]

**Table 1.** Search string testing in SCOPUS up to the year 2024

| Final search string | Search string in SCOPUS up to 2024  TITLE-ABS-KEY searches the title, abstract, and keywords | Number of results | Number of benchmark articles | Notes |
| --- | --- | --- | --- | --- |
|  | TITLE-ABS-KEY(hunt* OR overhunt* OR caza* OR caceria OR bushmeat OR "bush meat" OR poach OR game OR wildmeat OR "wild meat" OR "Carne salvaje" OR wildlife OR "wild life" AND mammal* OR mamífero OR "large vertebrates" AND peru* OR "Madre de Dios" OR amazonas OR ucayali OR loreto OR "San Martin" OR huanuco OR pasco OR junin OR huancavelica OR ayacucho OR apurimac OR cuzco OR puno OR tacna OR moquegua OR arequipa OR ica OR lima OR ancash OR "La Libertad" OR cajamarca OR lambayeque OR piura OR tumbes) | 160 | 11 of 13 | Test with mammal |
|  | TITLE-ABS-KEY(hunt* OR overhunt* OR bushmeat OR "bush meat" OR poach OR game OR wildmeat OR "wild meat" OR caza* OR caceria OR "cazar furtivamenta" OR "carne salvaje" OR "carne de animales silvestre" AND mammal* OR "large vertebrates" OR wildlife OR "wild life" OR species OR mamífero OR "grandes vertebrados" OR "vida silvestre" OR "vida salvaje" OR especie AND peru* OR "Madre de Dios" OR amazonas OR ucayali OR loreto OR "San Martin" OR huanuco OR pasco OR junin OR huancavelica OR ayacucho OR apurimac OR cuzco OR puno OR tacna OR moquegua OR arequipa OR ica OR lima OR ancash OR "La Libertad" OR cajamarca OR lambayeque OR piura OR tumbes) | 381 | 11 of 13 | Add the word species |
|  | TITLE-ABS-KEY(hunt* OR defaunation OR overhunt* OR bushmeat OR "bush meat" OR poach OR game OR wildmeat OR "wild meat" OR caza* OR caceria OR "cazar furtivamenta" OR "carne salvaje" OR "carne de animales silvestre" AND mammal* OR "large vertebrates" OR wildlife OR "wild life" OR species OR mamífero OR "grandes vertebrados" OR "vida silvestre" OR "vida salvaje" OR especie AND peru* OR "Madre de Dios" OR amazonas OR ucayali OR loreto OR "San Martin" OR huanuco OR pasco OR junin OR huancavelica OR ayacucho OR apurimac OR cuzco OR puno OR tacna OR moquegua OR arequipa OR ica OR lima OR ancash OR "La Libertad" OR cajamarca OR lambayeque OR piura OR tumbes) | 388 | 13 of 13 | Add word defaunation. No relevant papers added so removed it in the next addition below. |
|  | TITLE-ABS-KEY(hunt* OR overhunt* OR trap* OR bushmeat OR "bush meat" OR poach OR game OR wildmeat OR "wild meat" OR caza* OR caceria OR "cazar furtivamente" OR trampa* OR atrapan OR "carne salvaje" OR "carne de animales silvestre" AND mammal* OR "large vertebrates" OR wildlife OR "wild life" OR species OR mamífero OR "grandes vertebrados" OR "vida silvestre" OR "vida salvaje" OR especie AND peru* OR "Madre de Dios" OR amazonas OR ucayali OR loreto OR "San Martin" OR huanuco OR pasco OR junin OR huancavelica OR ayacucho OR apurimac OR cuzco OR puno OR tacna OR moquegua OR arequipa OR ica OR lima OR ancash OR "La Libertad" OR cajamarca OR lambayeque OR piura OR tumbes) | 879 | 13 of 13 | Adding trap |
|  | TITLE-ABS-KEY(hunt* OR overhunt* OR trap* OR bushmeat OR "bush meat" OR poach* OR game OR wildmeat OR "wild meat" OR caza* OR caceria OR "cazar furtivamente" OR trampa* OR atrapan OR "carne salvaje" OR "carne de animales silvestre" AND "large vertebrate*" OR wildlife OR "wild life" OR species OR "grandes vertebrados" OR "vida silvestre" OR "vida salvaje" OR especie AND peru* OR "Madre de Dios" OR amazonas OR ucayali OR loreto OR "San Martin" OR huanuco OR pasco OR junin OR huancavelica OR ayacucho OR apurimac OR cuzco OR puno OR tacna OR moquegua OR arequipa OR ica OR lima OR ancash OR "La Libertad" OR cajamarca OR lambayeque OR piura OR tumbes) | 874 | 13 of 13 | Only species, without mammals, birds, reptiles |
|  | TITLE-ABS-KEY(hunt* OR overhunt* OR trap* OR bushmeat OR "bush meat" OR poach* OR game OR wildmeat OR "wild meat" OR caza* OR caceria OR "cazar furtivamente" OR trampa* OR atrapan OR "carne salvaje" OR "carne de animales silvestre" AND reptile* OR bird* OR mammal* OR "large vertebrate*" OR wildlife OR "wild life" OR species OR reptil* OR ave* OR mamífero* OR "grandes vertebrados" OR "vida silvestre" OR "vida salvaje" OR especie AND peru* OR "Madre de Dios" OR amazonas OR ucayali OR loreto OR "San Martin" OR huanuco OR pasco OR junin OR huancavelica OR ayacucho OR apurimac OR cuzco OR puno OR tacna OR moquegua OR arequipa OR ica OR lima OR ancash OR "La Libertad" OR cajamarca OR lambayeque OR piura OR tumbes) | 1008 | 13 of 13 | Adding reptile, bird, mammal |
| Final search string | TITLE-ABS-KEY(hunt* OR overhunt* OR trap* OR bushmeat OR "bush meat" OR poach* OR game OR wildmeat OR "wild meat" OR caza* OR caceria OR "cazar furtivamente" OR trampa* OR atrapan OR "carne salvaje" OR "carne de animales silvestre" AND amphibian* OR reptile* OR chelonia* OR bird* OR cracid* OR mammal* OR "large vertebrate*" OR wildlife OR "wild life" OR species OR anfibio* OR reptil* OR quelonio* OR ave* OR crácido* OR mamífero* OR "grandes vertebrados" OR "vida silvestre" OR "vida salvaje" OR especie* AND peru* OR "Madre de Dios" OR amazonas OR ucayali OR loreto OR "San Martin" OR huanuco OR pasco OR junin OR huancavelica OR ayacucho OR apurimac OR cuzco OR puno OR tacna OR moquegua OR arequipa OR ica OR lima OR ancash OR "La Libertad" OR cajamarca OR lambayeque OR piura OR tumbes) | 1010 | 13 of 13 | Adding Chelonia and Cracids |

2. Final search string to be used in each database

SCOPUS

With Newcastle University login

In Command Line:

TITLE-ABS-KEY(hunt* OR overhunt* OR trap* OR bushmeat OR "bush meat" OR poach* OR game OR wildmeat OR "wild meat" OR caza* OR caceria OR "cazar furtivamente" OR trampa* OR atrapan OR "carne salvaje" OR "carne de animales silvestre" AND amphibian* OR reptile* OR chelonia* OR bird* OR cracid* OR mammal* OR "large vertebrate*" OR wildlife OR "wild life" OR species OR anfibio* OR reptil* OR quelonio* OR ave* OR crácido* OR mamífero* OR "grandes vertebrados" OR "vida silvestre" OR "vida salvaje" OR especie* AND peru* OR "Madre de Dios" OR amazonas OR ucayali OR loreto OR "San Martin" OR huanuco OR pasco OR junin OR huancavelica OR ayacucho OR apurimac OR cuzco OR puno OR tacna OR moquegua OR arequipa OR ica OR lima OR ancash OR "La Libertad" OR cajamarca OR lambayeque OR piura OR tumbes)

On page options:

Year: Range: 1957 (earliest paper in the system) – 2025

Web of Science Core Collection

With Newcastle University login

In Command Line:

Box 1 Drop down menu: Topic

Search box command line:

(hunt* OR overhunt* OR trap* OR bushmeat OR "bush meat" OR poach* OR game OR wildmeat OR "wild meat" OR caza* OR caceria OR "cazar furtivamente" OR trampa* OR atrapan OR "carne salvaje" OR "carne de animales silvestres" )AND (amphibian* OR reptile* OR chelonia* OR bird* OR cracid* OR mammal* OR "large vertebrate*" OR wildlife OR "wild life" OR species OR anfibio* OR reptil* OR quelonio* OR ave* OR crácido* OR mamífero* OR "grandes vertebrados" OR "vida silvestre" OR "vida salvaje" OR especie* )AND (peru* OR "Madre de Dios" OR amazonas OR ucayali OR loreto OR "San Martin" OR huanuco OR pasco OR junin OR huancavelica OR ayacucho OR apurimac OR cuzco OR puno OR tacna OR moquegua OR arequipa OR ica OR lima OR ancash OR "La Libertad" OR cajamarca OR lambayeque OR piura OR tumbes)

Scielo Citation Index

No login

In Command Line:

All indexes

(ab:((hunt* OR overhunt* OR trap* OR bushmeat OR "bush meat" OR poach* OR game OR wildmeat OR "wild meat" OR caza* OR caceria OR "cazar furtivamente" OR trampa* OR atrapan OR "carne salvaje" OR "carne de animales silvestres" )AND (amphibian* OR reptile* OR chelonia* OR bird* OR cracid* OR mammal* OR "large vertebrate*" OR wildlife OR "wild life" OR species OR anfibio* OR reptil* OR quelonio* OR ave* OR crácido* OR mamífero* OR "grandes vertebrados" OR "vida silvestre" OR "vida salvaje" OR especie* )AND (peru* OR "Madre de Dios" OR amazonas OR ucayali OR loreto OR "San Martin" OR huanuco OR pasco OR junin OR huancavelica OR ayacucho OR apurimac OR cuzco OR puno OR tacna OR moquegua OR arequipa OR ica OR lima OR ancash OR "La Libertad" OR cajamarca OR lambayeque OR piura OR tumbes))) OR (ti:((hunt* OR overhunt* OR trap* OR bushmeat OR "bush meat" OR poach* OR game OR wildmeat OR "wild meat" OR caza* OR caceria OR "cazar furtivamente" OR trampa* OR atrapan OR "carne salvaje" OR "carne de animales silvestres" )AND (amphibian* OR reptile* OR chelonia* OR bird* OR cracid* OR mammal* OR "large vertebrate*" OR wildlife OR "wild life" OR species OR anfibio* OR reptil* OR quelonio* OR ave* OR crácido* OR mamífero* OR "grandes vertebrados" OR "vida silvestre" OR "vida salvaje" OR especie* )AND (peru* OR "Madre de Dios" OR amazonas OR ucayali OR loreto OR "San Martin" OR huanuco OR pasco OR junin OR huancavelica OR ayacucho OR apurimac OR cuzco OR puno OR tacna OR moquegua OR arequipa OR ica OR lima OR ancash OR "La Libertad" OR cajamarca OR lambayeque OR piura OR tumbes)))

CAB Abstracts

With Newcastle University login

Title or abstracts searched:

TI ((hunt* OR overhunt* OR trap* OR bushmeat OR "bush meat" OR poach* OR game OR wildmeat OR "wild meat" OR caza* OR caceria OR "cazar furtivamente" OR trampa* OR atrapan OR "carne salvaje" OR "carne de animales silvestre") AND (amphibian* OR reptile* OR chelonia* OR bird* OR cracid* OR mammal* OR "large vertebrate*" OR wildlife OR "wild life" OR species OR anfibio* OR reptil* OR quelonio* OR ave* OR crácido* OR mamífero* OR "grandes vertebrados" OR "vida silvestre" OR "vida salvaje" OR especie*) AND (peru* OR "Madre de Dios" OR amazonas OR ucayali OR loreto OR "San Martin" OR huanuco OR pasco OR junin OR huancavelica OR ayacucho OR apurimac OR cuzco OR puno OR tacna OR moquegua OR arequipa OR ica OR lima OR ancash OR "La Libertad" OR cajamarca OR lambayeque OR piura OR tumbes))

OR AB ((hunt* OR overhunt* OR trap* OR bushmeat OR "bush meat" OR poach* OR game OR wildmeat OR "wild meat" OR caza* OR caceria OR "cazar furtivamente" OR trampa* OR atrapan OR "carne salvaje" OR "carne de animales silvestre") AND (amphibian* OR reptile* OR chelonia* OR bird* OR cracid* OR mammal* OR "large vertebrate*" OR wildlife OR "wild life" OR species OR anfibio* OR reptil* OR quelonio* OR ave* OR crácido* OR mamífero* OR "grandes vertebrados" OR "vida silvestre" OR "vida salvaje" OR especie*) AND (peru* OR "Madre de Dios" OR amazonas OR ucayali OR loreto OR "San Martin" OR huanuco OR pasco OR junin OR huancavelica OR ayacucho OR apurimac OR cuzco OR puno OR tacna OR moquegua OR arequipa OR ica OR lima OR ancash OR "La Libertad" OR cajamarca OR lambayeque OR piura OR tumbes))

Proximity search mode turned on

ProQuest Natural Science Collection

With Newcastle University login

Databases: Natural Science Collection

Abstract or Title searched

In Command Line:

abstract((hunt* OR overhunt* OR trap* OR bushmeat OR "bush meat" OR poach* OR game OR wildmeat OR "wild meat" OR caza* OR caceria OR "cazar furtivamente" OR trampa* OR atrapan OR "carne salvaje" OR "carne de animales silvestre") AND (amphibian* OR reptile* OR chelonia* OR bird* OR cracid* OR mammal* OR "large vertebrate*" OR wildlife OR "wild life" OR species OR anfibio* OR reptil* OR quelonio* OR ave* OR crácido* OR mamífero* OR "grandes vertebrados" OR "vida silvestre" OR "vida salvaje" OR especie*) AND (peru* OR "Madre de Dios" OR amazonas OR ucayali OR loreto OR "San Martin" OR huanuco OR pasco OR junin OR huancavelica OR ayacucho OR apurimac OR cuzco OR puno OR tacna OR moquegua OR arequipa OR ica OR lima OR ancash OR "La Libertad" OR cajamarca OR lambayeque OR piura OR tumbes))

OR title((hunt* OR overhunt* OR trap* OR bushmeat OR "bush meat" OR poach* OR game OR wildmeat OR "wild meat" OR caza* OR caceria OR "cazar furtivamente" OR trampa* OR atrapan OR "carne salvaje" OR "carne de animales silvestre") AND (amphibian* OR reptile* OR chelonia* OR bird* OR cracid* OR mammal* OR "large vertebrate*" OR wildlife OR "wild life" OR species OR anfibio* OR reptil* OR quelonio* OR ave* OR crácido* OR mamífero* OR "grandes vertebrados" OR "vida silvestre" OR "vida salvaje" OR especie*) AND (peru* OR "Madre de Dios" OR amazonas OR ucayali OR loreto OR "San Martin" OR huanuco OR pasco OR junin OR huancavelica OR ayacucho OR apurimac OR cuzco OR puno OR tacna OR moquegua OR arequipa OR ica OR lima OR ancash OR "La Libertad" OR cajamarca OR lambayeque OR piura OR tumbes))

No other filters used

LA Referencia

No login

In Command Line:

All fields (Todos los Campos)

(hunt* OR overhunt* OR trap* OR bushmeat OR "bush meat" OR poach* OR game OR wildmeat OR "wild meat" OR caza* OR caceria OR "cazar furtivamente" OR trampa* OR atrapan OR "carne salvaje" OR "carne de animales silvestre") AND (amphibian* OR reptile* OR chelonia* OR bird* OR cracid* OR mammal* OR "large vertebrate*" OR wildlife OR "wild life" OR species OR anfibio* OR reptil* OR quelonio* OR ave* OR crácido* OR mamífero* OR "grandes vertebrados" OR "vida silvestre" OR "vida salvaje" OR especie*) AND (peru* OR "Madre de Dios" OR amazonas OR ucayali OR loreto OR "San Martin" OR huanuco OR pasco OR junin OR huancavelica OR ayacucho OR apurimac OR cuzco OR puno OR tacna OR moquegua OR arequipa OR ica OR lima OR ancash OR "La Libertad" OR cajamarca OR lambayeque OR piura OR tumbes)

Language: Spanish

Then repeat search but with language: English

Renati (SENEDU)

In Command Line:

Search: All of Dspace

For: (hunt* OR overhunt* OR trap* OR bushmeat OR "bush meat" OR poach* OR game OR wildmeat OR "wild meat" OR caza* OR caceria OR "cazar furtivamente" OR trampa* OR atrapan OR "carne salvaje" OR "carne de animales silvestre") AND (amphibian* OR reptile* OR chelonia* OR bird* OR cracid* OR mammal* OR "large vertebrate*" OR wildlife OR "wild life" OR species OR anfibio* OR reptil* OR quelonio* OR ave* OR crácido* OR mamífero* OR "grandes vertebrados" OR "vida silvestre" OR "vida salvaje" OR especie*) AND (peru* OR "Madre de Dios" OR amazonas OR ucayali OR loreto OR "San Martin" OR huanuco OR pasco OR junin OR huancavelica OR ayacucho OR apurimac OR cuzco OR puno OR tacna OR moquegua OR arequipa OR ica OR lima OR ancash OR "La Libertad" OR cajamarca OR lambayeque OR piura OR tumbes)

No other filters

ALICIA

In Command Line:

Todos los Campos (All Fields)

(hunt* OR overhunt* OR trap* OR bushmeat OR "bush meat" OR poach* OR game OR wildmeat OR "wild meat" OR caza* OR caceria OR "cazar furtivamente" OR trampa* OR atrapan OR "carne salvaje" OR "carne de animales silvestre") AND (amphibian* OR reptile* OR chelonia* OR bird* OR cracid* OR mammal* OR "large vertebrate*" OR wildlife OR "wild life" OR species OR anfibio* OR reptil* OR quelonio* OR ave* OR crácido* OR mamífero* OR "grandes vertebrados" OR "vida silvestre" OR "vida salvaje" OR especie*) AND (peru* OR "Madre de Dios" OR amazonas OR ucayali OR loreto OR "San Martin" OR huanuco OR pasco OR junin OR huancavelica OR ayacucho OR apurimac OR cuzco OR puno OR tacna OR moquegua OR arequipa OR ica OR lima OR ancash OR "La Libertad" OR cajamarca OR lambayeque OR piura OR tumbes)

No other filters selected

Google Scholar:

Filters:

Language: English

Sort by relevance

Date: up to 2025

Search 1:

(hunt|overhunt|trap|bushmeat|"bush meat"|poach|game|wildmeat|"wild meat") (amphibian|reptile|chelonia|bird|cracid|mammal|"large vertebrate"|wildlife|"wild life"|species) peru

Search 2:

Language: Spanish

Sort by relevance

Date: up to 2025

(caza|caceria|"cazar furtivamente"|trampa|atrapan|"carne salvaje"|"carne de animales silvestres") (anfibio|reptil|quelonio|ave|crácido|mamífero|"grandes vertebrados"|"vida silvestre"|"vida salvaje"|especie) perú
