# Supplementary material for: What evidence exists on the drivers, ecological and socio-economic outcomes, and distribution of hunting in Peru: a systematic map protocol
Source: Environ Evid. 2026 Apr 30;15:7. doi: 10.1186/s13750-026-00386-9 (PMC13285517; doi:10.1186/s13750-026-00386-9)
Supplement: Supplementary file 4 — Additional file 4. Endnote deduplication steps. The duplication preference adjustment settings used in Endnote to remove duplicates. [file 13750_2026_386_MOESM4_ESM.docx]

EndNote deduplication steps

In EndNote version 21: Edit > preferences > duplicates

Round 1: select:

- Author
- Year
- Title

Criteria: Ignore spacing and punctuation

Primary reference criteria: Most complete

Round 2: select:

- Year
- Title

Criteria: Ignore spacing and punctuation

Primary reference criteria: Most complete

Round 3: select:

- Author
- Title

Criteria: Ignore spacing and punctuation

Primary reference criteria: Most complete

Round 4: select:

- Title

Criteria: Ignore spacing and punctuation

Primary reference criteria: Most complete

To differentiate Spanish and English titles:

Round 5: select:

- Author
- Year

Criteria: Ignore spacing and punctuation

Primary reference criteria: Most complete

Round 6: select:

- Author

Criteria: Ignore spacing and punctuation

Primary reference criteria: Most complete

Then, to remove duplicates due to extra spaces in title, reorder references by title in alphabetical order. Scroll and compare references and delete duplicates.
